# Does BMI Modify the Association between Vitamin D and Pulmonary Function in Children of the Mild Asthma Phenotype?

**DOI:** 10.3390/ijerph192416768

**Published:** 2022-12-14

**Authors:** Maria Michelle Papamichael, Catherine Itsiopoulos, Charis Katsardis, Dimitris Tsoukalas, Bircan Erbas

**Affiliations:** 1 Human Services & Sport, School of Allied Health, La Trobe University, Melbourne 3086, Australia; 2 European Institute of Molecular Medicine, 00198 Rome, Italy; 3 School of Health & Biomedical Sciences, RMIT University, Melbourne 3083, Australia; 4 Medical School, National & Kapodistrian University of Athens, 157 72 Athens, Greece; 5 School of Psychology & Public Health, La Trobe University, Melbourne 3086, Australia

**Keywords:** asthma, BMI, children, lung function, mild asthma, vitamin D, overweight

## Abstract

Vitamin D deficiency and obesity are global health problems that are associated with increased asthma risk in children. The purpose of this study was to investigate whether BMI modifies pulmonary function across vitamin D tertiles in pediatric asthma patients of the mild asthma phenotype. This cross-sectional study conducted from November 2016–September 2017 compared lung function variability as assessed by spirometry and nitric oxide in exhaled breath (FeNO) among 35 normal-weight and 26 overweight/obese Greek schoolchildren (5–12 years old) with mild asthma. Serum 25 (OH)D levels ≥ 30 ng/mL were defined as ‘sufficient’, 20–30 ng/mL ‘insufficient’, and <20 ng/mL ‘deficient’. Stratification by BMI category, linear regression showed positive associations between D, % FVC (β = 0.49, 95%CI: 0.05, 0.94), and % FEV_1_ (β = 0.48, 95%CI: −0.01, 0.95) in the normal-weight only, adjusted for age, sex, regular exercise, and medication. FEV_1_ was 10% higher in the normal-weight D-sufficient group compared to those D-deficient (β = 10.43, 95%CI: 0.54, 20.32). No associations were observed for the overweight/obese group or FeNO. In conclusion, BMI modified associations of vitamin D on airway mechanics in children of the mild asthma phenotype. Serum 25 (OH)D concentrations ≥ 30 ng/mL were associated with higher ventilation in central airways of normal-weight asthmatic children.

## 1. Introduction

Allergic disease, including asthma, is ranked the fourth most common chronic condition, representing a major global challenge, especially in developed countries, due to the continual growth in prevalence, severity, and adverse effect on the quality of life [1]. With respect to children, asthma a chronic inflammatory disease in the lungs is an urgent public health issue primarily in low to middle-income countries [2], with 14% of children ≥6 years of age at risk of having asthma symptoms [3]. Findings from the International Study of Asthma and Allergies in Childhood (ISAAC) highlighted that there exists considerable variation in trends of childhood asthma prevalence across countries, ranging from <10% in southern European countries up to 30% in Anglo-Saxon countries, attributed to age, country income, geographical diversity, and inconsistencies in asthma definition [4,5].

However, growing evidence demonstrates that asthma incidence in children is rapidly increasing in southern Mediterranean regions. Over the last 25 years, childhood asthma prevalence has increased 4-fold in Greece [6]. Recent data documented that the prevalence rate of asthma in Greek schoolchildren aged 5–12 years old has escalated from 6.1% in 2004 [7] to 31% in 2018, independent of socioeconomic and regional factors [8]. This is of clinical importance because asthma can lead to deficits in airway development and function in children that can persist into adulthood [9].

The etiology for the rise in asthma incidence during the past three decades is the complex interactions among genetic, environmental, and lifestyle factors, including obesity, dietary changes, and hypovitaminosis D as possible determinants [10]. Disturbingly, the latest findings of the World Health Organization’s European Childhood Obesity Surveillance Initiative (COSI), undertaken during 2015–2017, which measured trends in overweight and obesity among over 250,000 elementary schoolchildren from 36 countries, reported an upward trend in childhood overweight predominately in southern European countries. In fact, 40% of Greek children were overweight (42% of boys and 38% of girls) and up to 20% obese (20% of boys and 14% of girls) [11]. The concurrent increase in the prevalence of asthma and obesity in children has led investigators to speculate about a possible correlation between these two conditions [12]. Large cross-sectional and prospective studies involving adolescents and children show a relationship between high BMI and asthma [13,14,15]. A recent meta-analysis of data from 73,252 children (0–18 years) found that overweight and obesity increased asthma odds by 30% and wheezing odds by 90%, while overweight or obese girls had 34% increased odds of asthma than boys [12]. Nonetheless, there was substantial heterogeneity among studies with regard to age and diagnostic tools used to assess asthma, overweight, and obesity. In line with this meta-analysis, data from the Hellenic Action Plan for the Assessment, Prevention, and Treatment of Childhood Obesity, which was a school-based survey undertaken during 2012–2013 and involved 11,751 Greek schoolchildren aged 5–12 years old, reported that being overweight and obese increased asthma odds by 13% and 27%, respectively [8]. Obesity causes altered pulmonary mechanics, greater airway obstruction, and reduced lung volumes among children with asthma [16,17]. The exact mechanism involved is unknown, although it is believed that the obesity and asthma link involves metabolic dysregulation, systemic inflammation, and the mechanical load of truncal fat causing chest wall restriction [18].

From another perspective, there is growing interest in the scientific community about the role of vitamin D in disease pathogenesis. Vitamin D is a pleiotropic steroidal hormone with multiple biological effects that is obtained from skin exposure to sunlight (UV B rays) and dietary intake of fatty fish, fish oil, liver, egg yolk, fortified food products, and dietary supplements [19]. Besides contributing to bone growth and maintenance, vitamin D is known to play a critical role in adaptive and innate immune pathways that might be relevant in the prevention of asthma, reduction of asthma morbidity, and the modulation of exacerbation severity and inflammation [20]. Vitamin D deficiency is highly prevalent in Europe [21] and may be a risk factor for the development of obesity [22] and asthma. Observational studies have consistently found circulating levels of 25 (OH) D < 30 ng/mL among asthmatic children associated with poor asthma control, increased asthma severity [23], deficits in pulmonary function, [24] and increased airway inflammation [25]. Pooled analysis of cross-sectional data of 24,600 children and adolescents showed that the relative risk for the association between obesity and vitamin D deficiency was 1.41 [22]. Surprisingly, vitamin D deficiency is prevalent in children and adolescents residing in sunny regions of the Mediterranean basin including Greece [21]. Standardized data from infants and children in Greece reported 25 (OH)D levels < 20 ng/mL in 40.5% to 62.4% [26]. Furthermore, lower 25 (OH)D concentrations were reported in children with obesity compared to normal-weight peers [27]. The causes of low vitamin D in individuals with overweight and obesity are inconclusive, although it has been proposed that low UV exposure and vitamin D sequestration in body fat could lead to decreased bioavailability and tissue secretion [28] along with insulin resistance [22]. With weight gain, a positive energy balance causes adipocyte hypertrophy, fat deposition, hypoxia, and chronic stress. As a consequence, this causes dysregulation in adipokine secretion activating increased production of pro-inflammatory cytokines (IL-6, TNFα) [29]. These series of events set the milieu for systemic metabolic dysfunction and reduced insulin sensitivity. Alternatively, low serum vitamin D concentrations could increase macrophage and monocyte cell surface expression of Toll-like receptors (TLR-2, TLR4), thereby enhancing pro-inflammatory cytokine production of IL-6 and TNFα) [30]. This state of low-grade chronic inflammation could be one of the potential mechanisms interrelating vitamin D, obesity and asthma.

In light of the above, there is a paucity of data on the role of vitamin D in children of the obese-asthma phenotype [31], specifically in the Greek pediatric population. Extending from our research demonstrating that vitamin D sufficiency enhanced lung function in Greek mild asthmatic children following a Mediterranean diet enriched with fatty fish [32], we sought to assess the association of serum vitamin D levels, weight status, and lung function variability in these patients. We hypothesized that vitamin D deficiency (<20 ng/mL) is negatively associated with pulmonary function and contributes to increased airway inflammation in asthmatic children with overweight and obesity compared to normal-weight counterparts. Dietary data is beyond the scope of the present study and is available in previous publications [32,33].

## 2. Materials and Methods

### 2.1. Sample Recruitment

This cross-sectional study used baseline measurements from the Mediterranean diet enriched with fatty fish study of 72 asthmatic children aged 5–12 years attending an urban children’s asthma clinic in Athens, Greece, that was conducted from November 2016 to September 2017. The cohort design, data collection, and sample size calculation based on power analysis have been described in detail previously [33]. In brief, children were eligible to participate if their age ranged from 5–12 years, they had physician-diagnosed “mild asthma”, they were willing to consume fatty fish as part of the Mediterranean diet [34], and had no hospital admissions during data collection. According to the Global Initiative for Asthma (GINA), “mild asthma” is defined as ‘well-controlled asthma’ having less than twice weekly daytime asthma symptoms and need for medication, absence of nighttime symptoms, and limitations in daily activities due to asthma exacerbations [35]. Children were excluded from the study if they had chronic or severe asthma, were undergoing oral glucocorticoid therapy, suffered from food allergies (specifically to fish and seafood), gastroesophageal reflux disease, cystic fibrosis, or congenital respiratory disease [35] and were taking multivitamins or omega-3 fatty acid supplements. The parent study was conducted according to the principles outlined in the Declaration of Helsinki (1989) for human experiments, and the procedures were approved by the La Trobe University Human Ethics Committee, Australia (HEC 16-035). Written informed consent was obtained from parents before the recruitment of children.

### 2.2. Variables of Interest

#### 2.2.1. Pulmonary Function Tests

Pulmonary function testing was undertaken by trained personnel at the pediatric asthma clinic during routine medical consultations. Technical procedures, expiratory maneuvres acceptance criteria, and reproducibility were judged acceptable if they met or exceeded European Respiratory Society recommendations [36,37]. Height and weight were measured with a precision of 0.1 cm and 0.1 kg, respectively. Spirometry was performed using a portable spirometer (MIR Spirobank II, Medical International Research (MIR) Inc., New Berlin, WI, USA), and airway inflammation was measured from nitric oxide (NO) in exhaled breath using NO Breathe (Bedfont Inc., Maidstone, UK). In children, Forced Expiratory Volume in 1 s (FEV_1_), Forced Vital Capacity (FVC), and Peak Expiratory Flow (PEF) ≥ 80% predicted, the ratio of FEV_1_/FVC 75–85%, and Forced Expiratory Flow at 25–75% (FEF _25–75%_) > 65% including a z-score cut-off of −1.64 for symptomatic patients, represent normal lung function. [38] Fractional exhaled Nitric Oxide (FeNO) measurements ≥ 20 ppb is indicative of possible eosinophilic airway inflammation, poor asthma control, and medication adherence [37]. The primary outcomes of interest were the percent predicted FEV_1_, FVC, the ratio of FEV_1_/FVC, PEF, FEF _25–75%_ pre-bronchodilator administration, and FeNO.

#### 2.2.2. Overweight and Obesity Categorization

Overweight and obesity categories were defined using BMI (kg/m^2^) for children 2–18 years old as proposed by the International Obesity Task Force (IOTF) [39]. These thresholds are based on age and sex-specific percentile curves that correspond to the adult cut points of 25 and 30 kg/m^2^ for overweight and obesity at the age of 18 years. Data on weight status was available for 61 children (out of 72), and given that only eight children (*n* = 8) were categorized as obese, overweight and obese groups were combined for analysis.

#### 2.2.3. Quantification of Serum Vitamin D Concentrations

A single measurement of serum 25 (OH)D concentration was obtained using an Enzyme-Linked Immuno Assay (ELISA) method (Immunodiagnostic Systems (IDS), Bolton, Tyne & Wear, Bolton, UK) (Supplemental File S1) [40]. Serum 25 (OH)D is considered to be the best circulating biomarker of vitamin D metabolic status and reflects contributions from diet and sun exposure [41]. We classified serum 25 (OH)D levels ≥ 30 ng/mL as ‘sufficient’, 20 to 30 ng/mL ‘ insufficient’ and <20 ng/mL ‘deficient’ based on previous recommendations for bone health [41].

#### 2.2.4. Physical Activity

Physical activity level was evaluated by a question derived from the International Study on Asthma and Allergies in Childhood (ISAAC) Phase 3 Environmental Questionnaire [42] as follows: “How many times per week does your child engage in vigorous physical activity long enough to make him/her breathe hard?” Response options were “never or occasionally”, “once or twice per week”, and “three or more times a week”. Regular exercise was defined as, physical activity performed more than or equal to three times per week.

#### 2.2.5. Asthma Medication Therapy

Medication use in children was assessed by the following question: ”During the last month, has your child taken asthma medication?” Two response options were available: “Yes” or “No”.

### 2.3. Statistical Analyses

All data were analyzed using SPSS version 27 (IBM Corp. Armonk, NY, USA), and a two-tailed *p* < 0.05 was considered significant. Continuous variables were assessed for normality using the Shapiro–Wilks test and summarized descriptively as means, standard deviations, and percentages for categorical frequencies. Differences between and within groups were assessed using T-test and ANOVA with the Bonferroni correction for normally distributed variables, otherwise Mann–Whitney U test and Chi-Square tests. After stratification by BMI category (normal-weight versus overweight/obese), associations between vitamin D continuous independent variable and as the threshold for bone health (<20 ng/mL, 20–30 and ≥30 ng/mL) versus pulmonary function tests were assessed using linear regression models adjusted for age, sex, exercise and medication therapy based on the literature [10,43]. The effect size was reported by the unstandardized β-coefficient and 95% confidence interval (CI). In order to account for children’s dietary intake, we conducted a mediation analysis to explore whether the associations between vitamin D and spirometry were mediated by diet, as represented by the KIDMED score, a measure of children’s adherence to the Mediterranean dietary pattern [44].

## 3. Results

In the total population, of the 61 children with an average age of 8.0 (S.D 2.2) years old, 51% (*n* = 31) were female and almost half 42.6% (*n* = 26) were overweight/obese. Pulmonary function tests showed that children had normal lung function (as reflected by % predicted FEV_1_, FVC ≥ 80%, and the ratio of FEV_1_/FVC > 75–85%) and no airway inflammation (FeNO < 20 ppb). Regarding vitamin D status, only 9.8% (*n* = 6) of children were ‘D-sufficient’ (25 (OH) D ≥ 30 ng/mL), 60.7% (*n* = 37) ‘insufficient’ (25 (OH)D: 20–30 ng/mL) and 29.5 % (*n* = 18) were ‘deficient’ (25 (OH)D < 20 ng/mL). To our knowledge, no participants were taking vitamin D supplements. Population characteristics per sex are exhibited in (Supplemental Table S1). No differences were observed between males and females. Investigation of population and clinical characteristics according to BMI category showed no differences in vitamin D levels or spirometry except for FeNO, which was slightly lower in children of the overweight/obese group than in the normal-weight (mean (SD): 11.96 (15.46) vs. 15.74 (12.74); *p* = 0.045, respectively) (Table 1).

In contrast, differences were noted in lung function according to BMI category and serum vitamin D levels (Table 2). Spirometry indices %FVC and %FEV_1_ were significantly lower in the normal-weight D-deficient group than in the overweight/obese counterparts and FeNO higher [D < 20 ng/mL normal vs. overweight/obese mean (SD): %FVC 88.36 (10.73) vs. 102.00 (12.40), *p* = 0.025; %FEV_1_ 92.55 (10.38) vs. 105.00 (12.11), *p* = 0.033; 14.91 (16.73) vs. 4.14 (3.58) ppb, *p* = 0.045, respectively]. Within-group differences showed that, for the normal-weight group, as vitamin D increased from deficient to sufficient, there was an increasing trend for FVC and FEV_1_ (*p* = 0.028, 0.036, respectively), while no differences were observed for children in the overweight/obese group (*p* = 0.31, 0.36, respectively). 

### Regression Model

Stratification of data according to BMI category (normal-weight vs. overweight/obese) showed that in the normal-weight group there were significant positive associations between vitamin D and %FVC (β = 0.49, 95%CI: 0.05, 0.94; P_adj_ = 0.031) and %FEV_1_ (β = 0.48, 95%CI: −0.01, 0.95; P_adj_ = 0.047), after adjusting for age, sex, regular exercise and medication therapy (Table 3).

Similar trends were observed when entering vitamin D levels as the independent variables (Table 4). In the adjusted analysis, %FEV_1_ was significantly higher by approximately 10% in the normal-weight vitamin D-sufficient as compared to those D-deficient (β = 10.43, 95%CI: 0.54, 20.32; P_adj_ = 0.040). In both analyses, no associations were noted for the overweight/obese group or FeNO. The mediation analysis revealed no association between vitamin D and the KIDMED score in either the normal-weight or overweight/obese groups (normal-weight *p* = 0.28; overweight/obese *p* = 0.73). In addition, the KIDMED score was not associated with FVC or FEV_1_ in either BMI group (FVC: normal-weight *p* = 0.73; overweight/obese *p* = 0.84); (FEV_1_: normal-weight *p* = 0.87; overweight/obese *p* = 0.74).

## 4. Discussion

Vitamin D plays a vital role in airway physiology. In the current study, we endeavored for the first time to elucidate the relationship among serum vitamin D concentrations, adiposity, and respiratory function in a Hellenic pediatric population suffering from mild asthma. We found that the prevalence of suboptimal vitamin D concentrations (25 (OH)D < 30 ng/mL) was high in our sample of asthmatic children (90%) independent of BMI category, indicating a link between vitamin D levels and the asthmatic state [45]. This is in line with a previous meta-analysis undertaken by Jat et al. demonstrating that asthmatic children were 3.41 times more likely to be vitamin D deficient and 2.34 times more likely insufficient compared to non-asthmatic counterparts [45]. Furthermore, in the present study, differences were noted in lung function according to BMI category and per vitamin D tertile. Spirometry indices %FVC and %FEV_1_ were significantly lower in the normal-weight D-deficient group than in the overweight/obese counterparts and FeNO was higher. Specifically, for the normal-weight group, as vitamin D concentrations increased from deficient to sufficient, there was an increasing trend for FVC and FEV_1_. Notably, regression models revealed positive associations between vitamin D and markers of central airway function (FVC and FEV_1_) in normal-weight children of the mild asthma phenotype. In fact, in normal-weight children, %FEV_1_ was 10% higher in those D-sufficient compared to vitamin D-deficient children, after adjusting for age, sex, regular exercise, and medication therapy. Contrastingly, no associations were observed in patients with overweight/obesity which rejects our original hypothesis and shows that the relationship is more complex than we first understood. Consistent with our findings, a recent meta-analysis of 27 observational studies reported that children that were D-deficient or D-insufficient had significantly poorer lung function as reflected by %FEV_1_ than those D-sufficient [46]. In reference to the Mediterranean region, positive relationships were observed between serum vitamin D levels and FVC and FEV_1_ in 75 Italian children (5–11 years) with asthma, thereby supporting the credibility of our findings [47].

Vitamin D has been referred to as having pleiotropic properties that regulate the immune response, dampen inflammation, and influence airway remodeling, a pathological feature of asthma [48]. Molecular research has shown that vitamin D receptors (VDRs) are widely distributed in respiratory epithelial cells, airway smooth muscle cells, and immune cells (B and T cells, macrophages, and monocytes), thus suggesting that vitamin D may have a potential role in asthma mediated by immunomodulatory function [48]. The active form of vitamin D (1, 25 (OH)_2_D) exerts its physiological effects by binding to VDRs and co-activation of VDR with the retinoid X receptor (RXR) promoting vitamin-D regulated gene transcription [48], suppression of IL-4 mediated expression of pro-inflammatory cytokines IL-13 and IL-17, and stimulate the secretion of T regulatory cells (T reg) as well as the anti-inflammatory cytokine (IL-10) [48]. Additionally, 1, 25 (OH)_2_D acts on T cells, causing a shift from the Th1 phenotype to the less inflammatory Th2 [48]. In the context of airway remodeling, a feature of asthma, activated 1, 25 (OH)_2_D can suppress fibroblast proliferation and expression of pro-inflammatory chemokines (TNF-α, TGF-β) from smooth muscle cells including inhibit matrix metalloproteinases which are involved in the digestion of the extracellular matrix [48]. Therefore, this scenario suggests that having sufficient serum concentrations of 25 (OH) D ≥ 30 ng/mL could attenuate inflammation-induced airway remodeling in smooth muscle cells of normal-weight pediatric asthma patients and consequently result in improved airflow and ventilation as was observed in our study. Achievement of vitamin D levels ≥ 30 ng/mL in high-risk pediatric patients with suboptimal levels via sun exposure, dietary modification, and supplementation could optimize lung function and attenuate the asthma burden in children. Just 5–30 min of outdoor play twice weekly (depending on the time of day, season, latitude and skin pigmentation) and regular consumption of vitamin-D rich foods such as fatty fish, liver, egg yolk, and fortified food products provide adequate vitamin D for overall health benefits [43] and could potentially ameliorate airway inflammation in asthmatic children. Recently, we demonstrated in a six-month clinical trial that a weekly intake of fatty fish reduced airway inflammation in children with mild asthma [33]. This raises the possibility of vitamin D as a promising non-pharmacological alternative or adjunct therapy for improving pulmonary mechanics, given that medication adherence is poor in asthmatic children [49]. Clearly, more studies are needed.

Contrastingly, in the regression analysis, null associations were found between vitamin D and spirometry indices in children with overweight/obesity. The absence of a protective effect of sufficient vitamin D concentrations on lung function in children of the overweight/obese group might be ascribed to the accumulation of vitamin D in adipose tissue, which leads to reduced bioavailability, metabolism, and absorption [28]. Along these lines, given that adipose tissue expresses VDR protein and enzymes involved in vitamin D metabolism, then feasibly, vitamin D resistance due to VDR dysfunction could also be a limiting factor [50]. Apart from this, another plausible explanation accounting for the observed non-associations in our overweight/obese group could be due to low statistical power since only eighteen children were categorized as overweight and eight, as obese. Lautenbacher et al. documented lower FEV_1_ and Functional Residual Capacity (FRC) among Hispanic/African American pediatric asthma patients with obesity and D-deficient than sufficient and insufficient peers, respectively [31]. Notably, our results emphasize the importance of maintaining lean body weight and optimal vitamin D status to improve lung function indices [51]. Nevertheless, our findings need to be replicated in other studies of mild asthmatic children who are overweight or obese.

### Strengths and Limitations

We showed that pediatric asthma patients residing in the Mediterranean region could be at risk of developing hypovitaminosis D [23]. To our knowledge, overall, this is the second study to investigate the role of vitamin D in relation to lung function in children of the obese-asthma phenotype [31] and the first conducted in European populations, thus adding new evidence to the limited database. Another strength of this study is the inclusion of the airway inflammatory index marker FeNO as an adjunct to the gold standard of lung function tests, spirometry, to obtain a complete clinical evaluation of airway status. Consistent with the majority of studies investigating the effect of body weight on lung function in asthmatic children [52,53], BMI was used as an acceptable proxy measure of overall adiposity, which can be calculated with a high degree of reliability and accuracy. Nonetheless, BMI is not a direct measure of body fat, and it is possible that the distribution of body fat in children may have a unique influence on lung function indices and is worth investigating in future studies using alternative methods that account for fat distribution such as waist circumference (a measure of abdominal obesity), body composition using DEXA, skinfold thickness, or impedance [54].

Despite the strengths of our study, some caveats deserve mention. Considering the cross-sectional design of our study, we cannot draw conclusions about causality. The limited number of participants in the present study makes interpretation difficult because of the low sample size [55]. Additionally, we did not have a control group of non-asthmatic patients as a comparison, and this merits further exploration in another study. Vitamin D levels were measured at only one-time point from November to January, when cutaneous vitamin D production is lowest, [43] and we did not consider seasonal changes, sunlight exposure, and vitamin D supplementation as potential confounding factors. One more shortcoming, although the gold standard for 25 (OH)D determination from blood samples is Liquid Chromatography-Mass Spectroscopy (LC-MS), ELISA is cheaper and does not require specialized technicians. One disadvantage is that immunoassay methods show bias and increased variability relative to LC-MS [56]. Nevertheless, the use of total 25 (OH)D is common in epidemiological studies because it is stable under a variety of laboratory conditions and in specimens for long-term storage [56].

## 5. Conclusions

Vitamin D deficiency and obesity are global health problems requiring attention in public health that are associated with increased asthma risk in children. In this study, BMI modified associations of vitamin D on airway mechanics in children of the mild asthma phenotype. Serum 25 (OH)D concentrations ≥ 30 ng/mL were associated with higher ventilation in the central airways of normal-weight asthmatic children. Achieving 25 (OH)D concentrations above 30 ng/mL in mild asthmatic patients may be a viable strategy for optimizing lung function, targeting airway inflammation, and remodeling in the treatment of pediatric asthma. More studies in mild asthmatic children who are overweight and vitamin D-insufficient are needed to replicate our findings.

## Figures and Tables

**Table 1:** Population and clinical characteristics according to BMI category.

	BMI Category		
	Normal (*n* = 35)	Overweight/Obese (*n* = 26)	
Characteristic	Mean (SD)	Mean (SD)	P ^a^
Male% (*n*)	51.43% (18/35)	46.15% (12/26)	
Female% (*n*)	48.57% (17/35)	53.85% (14/26)	
Patient’s Age (years)	7.86 (2.43)	8.23 (2.03)	0.37 ^b^
BMI (kg/m^2^)	16.54 (1.84)	21.63 (3.67) ^d^	**<0.001 ^b^**
**PFTs**			
% FVC *	93.89 (9.65)	97.85 (9.56)	0.12
% FEV_1_	96.49 (10.19)	100.73 (9.14)	0.10
% FEV_1_/FVC	102.06 (6.78)	102.27 (5.14)	1.00 ^b^
% PEF	92.91 (18.86)	92.27 (13.66)	0.69 ^b^
% FEF _25–75%_	99.46 (20.54)	105.50 (18.03)	0.24
FeNO (ppb)	15.74 (12.74)	11.96 (15.46)	**0.04**
Vitamin D (ng/mL)	24.05 (7.89)	21.92 (6.10)	0.18
Regular exercise % (*n*) ≥3 times/week	51.43% (18/35)	46.15% (12/26)	0.68 ^c^
Medication Therapy Yes	82.86% (29/35)	80.77% (21/26)	0.83 ^c^
25 (OH) D levels based on bone health			
<20 ng/mL (deficient) % (*n*)	31.43% (11/35)	26.92% (7/26)	0.32 ^c^
20–30 ng/mL (insufficient) % (*n*)	54.29% (19/35)	69.23% (18/26)	
≥30 ng/mL (sufficient) % (*n*)	14.29% (5/35)	3.85% (1/26)	

In bold text statistically significant *p*-values. N = 61; * % predicted spirometric indices pre-bronchodilator administration; ^a^ P—*p* value calculated using *t*-test, ^b^ Mann–Whitney, ^c^ Chi Square test; ^d^ Based on IOTF cut-off points for overweight/obesity [39]; *p*-value significant at 0.05; Key: PFTs—Pulmonary Function Tests; BMI—Body Mass Index; FEV_1_—Forced Expiratory Volume in 1 s; FVC—Forced Vital Capacity, FEV_1_/FVC—ratio of Forced Expiratory Volume in 1 s and Forced Vital Capacity, PEF—Peak Expiratory Flow, FEF _25–75%_—Forced Expiratory Flow at 25–75% of the pulmonary volume, FeNO—Fractional exhaled Nitric Oxide.

**Table 2:** Population characteristics according to BMI category and by vitamin D level.

	D< 20 ng/mL Deficient			D 20–30 ng/mL Insufficient		
	Normal (*n* = 11)	Overweight/Obese (*n* = 7)		Normal (*n* = 19)	Overweight/Obese (*n* = 18)	
Variable	Mean (SD)	Mean (SD)	P ^a^	Mean (SD)	Mean (SD)	P ^a^
Male	36.36% (4)	28.57% (2)	0.73 ^c^	52.63% (10)	50.00% (9)	0.87 ^c^
Female	63.64% (7)	71.43% (5)		47.37% (9)	50.00% (9)	
Age (years)	7.82 (2.68)	8.00 (2.45)	0.85 ^b^	7.89 (2.40)	8.33 (1.97)	0.42 ^b^
Regular exercise ≥3 x/week % (*n*)	27.27% (3)	57.14% (4)	0.20 ^c^	57.89% (11)	38.89% (7)	0.25 ^c^
Medication therapy Yes % (*n*)	90.91% (10)	85.71% (6)	0.73 ^c^	78.95% (15)	77.78% (14)	0.93 ^c^
**PFTs**						
%FVC *	88.36 (10.73)	102.00 (12.40)	**0.025 ^a^**	95.16 (7.97)	96.72 (8.19)	0.56 ^a^
%FEV_1_	92.55 (10.38)	105.00 (12.11)	**0.033 ^a^**	96.16 (8.74)	99.28 (7.78)	0.26 ^a^
% FEV_1_/FVC	103.45 (5.34)	102.43 (2.99)	0.53 ^b^	100.58 (7.46)	101.94 (5.86)	0.63 ^b^
% PEF	88.73 (21.08)	96.86 (19.20)	0.28 ^b^	93.58 (18.87)	90.56 (11.51)	0.82 ^b^
% FEF _25–75%_	94.36 (14.61)	107.86 (14.69)	0.07	100.37 (23.72)	104.17 (19.86)	0.60 ^a^
FeNO (ppb)	14.91 (16.73)	4.14 (3.58)	**0.045 ^b^**	15.47 (10.04)	14.78 (17.67)	0.29 ^b^

In bold text statistically significant *p*-values; *n* = 61; * Spirometric indices %predicted pre-bronchodilator administration; ^a^ *p*-value estimated using T-test, ^b^ Mann–Whitney U test, ^c^ Chi-Square test; Key: PFTs—Pulmonary Function Tests, FEV_1_—Forced Expiratory Volume in 1 s, FVC—Forced Vital Capacity, FEV_1_/FVC—ratio of Forced Expiratory Volume in 1 s and Forced Vital Capacity, PEF—Peak Expiratory Flow, FEF _25–75%_—Forced Expiratory Flow at 25–75% of the pulmonary volume, FeNO—Fractional exhaled Nitric Oxide.

**Table 3:** Associations between vitamin D and pulmonary function tests by BMI category from the crude and adjusted linear regression analysis.

					BMI Category			
	Normal				Overweight/Obese			
PFT Parameters	Crude		Adjusted		Crude		Adjusted	
	β (95%CI)	P	β (95%CI)	P_adj_	β (95%CI)	P	β (95%CI)	P_adj_
% FVC *	**0.50 (0.10, 0.89)**	**0.015**	**0.49 (0.05, 0.94)**	**0.031**	−0.47 (−1.10, 0.15)	0.13	−0.48 (−1.20, 0.23)	0.17
% FEV_1_	0.43 (−0.00, 0.86)	0.05	**0.48 (0.01, 0.95)**	**0.047**	−0.40 (−1.01, 0.21)	0.19	−0.39 (−1.10, 0.32)	0.26
% FEV_1_/FVC	−0.07 (−0.37, 0.23)	0.63	−0.04 (−0.39, 0.31)	0.81	−0.06 (−0.30, 0.41)	0.74	−0.08 (−0.30, 0.46)	0.65
% PEF	0.47 (−0.36, 1.30)	0.26	0.33 (−0.63, 1.28)	0.49	−0.34 (−1.27, 0.59)	0.46	−0.50 (−1.57, 0.58)	0.34
% FEF _25–75%_	0.30 (−0.61, 1.22)	0.51	0.44 (−0.61, 1.49)	0.39	−0.08 (−1.33, 1.16)	0.89	−0.10 (−1.52, 1.31)	0.88
FeNO	0.11 (−0.45, 0.69)	0.68	0.28 (−0.35, 0.93)	0.37	0.47 (−0.57, 1.52)	0.36	0.81 (−0.31, 1.94)	0.15

In bold text statistically significant *p*-values; *n* (normal weight) = 35; *n* (overweight/obese) = 26; * Percent predicted pre-bronchodilator administration; Dependent variable—Pulmonary function test indices; Independent variable −25 (OH)D; β—Unstandardized beta coefficient; 95%CI—95% confidence interval; P_adj_—*p*-value from the multivariate linear regression model adjusted for age, sex, regular exercise, medication therapy; Key: PFT—Pulmonary Function Test, FEV_1_—Forced Expiratory Volume in 1 s, FVC—Forced Vital Capacity, FEV_1_/FVC—ratio of Forced Expiratory Volume in 1 s and Forced Vital Capacity, PEF—Peak Expiratory Flow, FEF _25–75%_—Forced Expiratory Flow at 25–75% of the pulmonary volume, FeNO—Fractional exhaled Nitric Oxide.

**Table 4:** Associations between vitamin D levels and pulmonary function tests by BMI category.

	Normal Weight				Overweight/Obese			
PFT Indices/ Vitamin D Levels	Crude		Adjusted		Crude		Adjusted	
	β (95%CI)	P	β (95%CI)	P_adj_	β (95%CI)	P	β (95%CI)	P_adj_
%FVC *								
D < 20	−6.79 (−13.66, 0.07)	0.05	−6.90 (−14.34, 0.54)	0.07	5.28 (−3.45, 14.00)	0.22	5.23 (−4.46, 14.93)	0.27
20 < D < 30								
D ≥ 30	6.04 (−3.06, 15.15)	0.19	5.71 (−3.95, 15.37)	0.24	−7.72 (−27.84, 12.40)	0.43	−6.09 (−28.82, 16.63)	0.58
%FEV_1_								
D < 20	−3.16 (−10.92, 3.69)	0.32	−4.72 (−12.34, 2.90)	0.21	5.72 (−2.67, 14.12)	0.17	5.98 (−3.60, 15.55)	0.21
20 < D< 30								
D ≥ 30	10.24 (0.55, 19.93)	**0.039**	10.43 (0.54, 20.32)	**0.040**	−2.28 (21.64, 17.09)	0.81	−1.03 (−23.49, 21.41)	0.92
%FEV_1_/FVC								
D < 20	2.88 (−2.35, 8.10)	0.27	2.04 (−3.69, 7.78)	0.47	0.48 (−4.36, 5.33)	0.84	0.67 (−4.43, 5.77)	0.79
20 < D < 30								
D ≥ 30	4.02 (−2.91, 10.95)	0.25	4.29 (−3.17, 11.74)	0.25	5.06 (−6.13, 16.24)	0.36	4.49 (−7.48, 16.46)	0.44
%PEF								
D < 20	−4.85 (−19.58, 9.88)	0.51	−3.39 (−19.70, 12.92)	0.67	6.30 (−6.53, 19.14)	0.32	8.29 (−6.09, 22.67)	0.24
20 < D < 30								
D ≥ 30	6.02 (−13.52, 25.57)	0.53	4.29 (−16.88, 25.46)	0.68	0.44 (−29.16, 30.05)	0.97	−1.25 (−34.95, 32.46)	0.94
%FEF _25–75%_								
D < 20	−6.00 (−22.00, 9.99)	0.45	−9.01 (−26.52, 8.50)	0.30	3.69 (−13.49, 20.88)	0.66	3.81 (−15.30, 22.92)	0.68
20 < D < 30								
D ≥ 30	6.83 (−14.39, 28.05)	0.52	8.26 (−14.47, 30.99)	0.46	8.83 (−30.81, 48.48)	0.65	4.48 (−40.31, 49.27)	0.84
FeNO								
D < 20	−0.56 (−10.65, 9.52)	0.91	−2.65 (−13.53, 8.24)	0.62	−10.63 (−24.74, 3.47)	0.13	−11.89 (−26.84, 3.05)	0.11
20 < D < 30								
D ≥ 30	3.13 (−10.25, 16.51)	0.64	5.25 (−8.88, 19.38)	0.45	1.22 (−31.30, 33.75)	0.94	5.16 (−29.86, 40.19)	0.76

*n* (normal weight) = 35; *n*(overweight/obese) = 26; * Percent predicted pre-bronchodilator administration; In bold statistically significant *p*-values; Dependent variable—Pulmonary Function Test (PFT) indices; Independent variable—Vitamin D levels (D < 20, 20–30 and ≥30 ng/mL); β—Unstandardized beta coefficient; 95%CI—95% confidence interval; P—*p* value from the crude analysis; P_adj_—Multivariate regression model adjusted for age, sex, regular exercise and medication therapy; Key: PFT—Pulmonary Function Test, D—Vitamin D, FEV_1_—Forced Expiratory Volume in 1 s, FVC—Forced Vital Capacity, FEV_1_/FVC—ratio of Forced Expiratory Volume in 1 s and Forced Vital Capacity, PEF—Peak Expiratory Flow, FEF _25–75%_ -Forced Expiratory Flow at 25–75% of the pulmonary volume, FeNO—Fractional exhaled Nitric Oxide.

## Data Availability

All data generated or analyzed during this study are included in this published article and its supplementary information files.

## Supplementary Materials

The following supporting information can be downloaded at: https://www.mdpi.com/article/10.3390/ijerph192416768/s1, File S1: Methods, Table S1: Population characteristics per sex. References [57,58,59] are cited in Supplementary Materials.

## References

[1] Enilari O., Sinha S. (2019). The Global Impact of Asthma in Adult Populations. Ann. Glob. Health.

[2] Dharmage S.C., Perret J.L., Custovic A. (2019). Epidemiology of Asthma in Children and Adults. Front. Pediatr..

[3] GAN The Global Asthma Report 2014. http://isaac.auckland.ac.nz/resources/Global_Asthma_Report_2014.pdf.

[4] Asher M.I., Rutter C.E., Bissell K., Chiang C.-Y., El Sony A., Ellwood E., Ellwood P., García-Marcos L., Marks G.B., Morales E. (2021). Worldwide trends in the burden of asthma symptoms in school-aged children: Global Asthma Network Phase I cross-sectional study. Lancet.

[5] Lai C., Beasley R., Crane J., Foliaki S., Shah J., Weiland S. (2009). Global variation in the prevalence and severity of asthma symptoms: Phase three of the International Study of Asthma and Allergies in Childhood (ISAAC). Thorax.

[6] Anthracopoulos M.B., Liolios E., Panagiotakos D.B., Triantou K., Priftis K.N. (2007). Prevalence of asthma among schoolchildren in Patras, Greece: Four questionnaire surveys during 1978–2003. Arch. Dis. Child.

[7] Sichletidis L., Chloros D., Tsiotsios I., Gioulekas D., Kyriazis G., Spyratos D., Charalambidou O., Goutsikas S. (2004). The prevalence of allergic asthma and rhinitis in children of Polichni, Thessaloniki. Allergol. Immunopathol..

[8] Karachaliou F., Vlachopapadopoulou E., Psaltopoulou T., Manios Y., Koutsouki D., Bogdanis G., Carayianni V., Sergentanis T., Hatzakis A., Michalacos S. (2020). Prevalence of asthma symptoms and association with obesity, sedentary lifestyle and sociodemographic factors: Data from the Hellenic National Action Plan for the assessment, prevention and treatment of childhood obesity (MIS301205). J. Asthma.

[9] McGeachie M.J., Yates K.P., Zhou X., Guo F., Sternberg A.L., Van Natta M.L., Wise R.A., Szefler S.J., Sharma S., Kho A.T. (2016). Patterns of Growth and Decline in Lung Function in Persistent Childhood Asthma. N. Engl. J. Med..

[10] Beasley R., Semprini A., Mitchell E.A. (2015). Risk factors for asthma: Is prevention possible?. Lancet.

[11] Spinelli A., Buoncristiano M., Nardone P., Starc G., Hejgaard T., Júlíusson P.B., Fismen A.-S., Weghuber D., Musić Milanović S., García-Solano M. (2021). Thinness, overweight, and obesity in 6- to 9-year-old children from 36 countries: The World Health Organization European Childhood Obesity Surveillance Initiative—COSI 2015–2017. Obes. Rev..

[12] Deng X., Ma J., Yuan Y., Zhang Z., Niu W. (2019). Association between overweight or obesity and the risk for childhood asthma and wheeze: An updated meta-analysis on 18 articles and 73252 children. Pediatr. Obes..

[13] Chih A.-H., Chen Y.-C., Tu Y.-K., Huang K.-C., Chiu T.-Y., Lee Y.L.J.E.R.J. (2016). Mediating pathways from central obesity to childhood asthma: A population-based longitudinal study. Eur. Respir. J..

[14] den Dekker H.T., Ros K.P.I., de Jongste J.C., Reiss I.K., Jaddoe V.W., Duijts L. (2017). Body fat mass distribution and interrupter resistance, fractional exhaled nitric oxide, and asthma at school-age. J. Allergy Clin. Immunol..

[15] Okubo Y., Nochioka K., Hataya H., Sakakibara H., Terakawa T., Testa M.J.T.J.o.A., Practice C.I.I. (2016). Burden of obesity on pediatric inpatients with acute asthma exacerbation in the United States. J. Allergy Clin. Immunol. Pract..

[16] Spathopoulos D., Paraskakis E., Trypsianis G., Tsalkidis A., Arvanitidou V., Emporiadou M., Bouros D., Chatzimichael A. (2009). The effect of obesity on pulmonary lung function of school aged children in Greece. Pediatr. Pulmonol..

[17] Vitiello L., Picariello M., Vitale C., Maglio A., Cerrone F., Schirò M., Vajro P., Vatrella A. (2018). Obesity and respiratory function impairment in asthmatic and non asthmatic children. Pediatrics.

[18] Vijayakanthi N., Greally J.M., Rastogi D. (2016). Pediatric Obesity-Related Asthma: The Role of Metabolic Dysregulation. Pediatrics.

[19] Hall S.C., Agrawal D.K. (2017). Vitamin D and Bronchial Asthma: An Overview of Data From the Past 5 Years. Clin. Ther..

[20] Pfeffer P.E., Hawrylowicz C.M. (2018). Vitamin D in Asthma: Mechanisms of Action and Considerations for Clinical Trials. Chest.

[21] Lips P., Cashman K.D., Lamberg-Allardt C., Bischoff-Ferrari H.A., Obermayer-Pietsch B., Bianchi M.L., Stepan J., El-Hajj Fuleihan G., Bouillon R. (2019). Current vitamin D status in European and Middle East countries and strategies to prevent vitamin D deficiency: A position statement of the European Calcified Tissue Society. J. Eur. J. Endocrinol..

[22] Fiamenghi V.I., Mello E.D. (2021). Vitamin D deficiency in children and adolescents with obesity: A meta-analysis. J. Pediatr..

[23] Havan M., Razi C.H., Bulus A.D., Köksal A.O., Andıran N. (2017). Effects of 25 hydroxy vitamin D levels on the severity and asthma control in school age asthma patients. Arch. Argent. Pediatr..

[24] Han Y.Y., Forno E., Celedón J.C. (2017). Vitamin D Insufficiency and Asthma in a US Nationwide Study. J. Allergy Clin. Immunol. Pract..

[25] Miraglia Del Giudice M., Allegorico A., Grandone A., Galdo F., Cuppari C., Capasso M., Maiello N. (2015). Vitamin D and bronchial inflammation in asthmatic children. J. Biol. Regul. Homeost. Agents.

[26] Cashman K.D., Dowling K.G., Skrabakova Z., Gonzalez-Gross M., Valtuena J., De Henauw S., Moreno L., Damsgaard C.T., Michaelsen K.F., Molgaard C. (2016). Vitamin D deficiency in Europe: Pandemic?. Am. J. Clin. Nutr..

[27] Moschonis G., Androutsos O., Hulshof T., Dracopoulou M., Chrousos G.P., Manios Y. (2018). Vitamin D insufficiency is associated with insulin resistance independently of obesity in primary schoolchildren. The healthy growth study. Pediatr. Diabetes.

[28] Wortsman J., Matsuoka L.Y., Chen T.C., Lu Z., Holick M.F. (2000). Decreased bioavailability of vitamin D in obesity. Am. J. Clin. Nutr..

[29] Zakharova I., Klimov L., Kuryaninova V., Nikitina I., Malyavskaya S., Dolbnya S., Kasyanova A., Atanesyan R., Stoyan M., Todieva A. (2019). Vitamin D Insufficiency in Overweight and Obese Children and Adolescents. Front. Endocrinol..

[30] Alsharairi N.A. (2020). Serum 25-hydroxyvitamin D is associated with obesity and metabolic parameters in US children. Public Health Nutr..

[31] Lautenbacher L.A., Jariwala S.P., Markowitz M.E., Rastogi D. (2016). Vitamin D and pulmonary function in obese asthmatic children. Pediatr. Pulmonol..

[32] Papamichael M.M., Itsiopoulos C., Lambert K., Katsardis C., Tsoukalas D., Erbas B. (2020). Sufficient vitamin D status positively modified ventilatory function in asthmatic children following a Mediterranean diet enriched with fatty fish intervention study. Nutr. Res..

[33] Papamichael M.M., Katsardis C., Lambert K., Tsoukalas D., Koutsilieris M., Erbas B., Itsiopoulos C. (2019). Efficacy of a Mediterranean diet supplemented with fatty fish in ameliorating inflammation in paediatric asthma: A randomised controlled trial. J. Hum. Nutr. Diet..

[34] Papamichael M.M., Katsardis C., Tsoukalas D., Erbas B., Itsiopoulos C. (2018). A Clinical Trial of Mediterranean Diet Enriched with Fatty Fish in Pediatric Asthma: Study Protocol. J. Pharm. Pharm..

[35] GINA (2020). Global Initiative for Asthma: Global Strategy for Asthma Management and Prevention. https://ginasthma.org.

[36] Miller M.R., Hankinson J., Brusasco V., Burgos F., Casaburi R., Coates A., Crapo R., Enright P., van der Grinten C.P.M., Gustafsson P. (2005). Standardisation of spirometry. Eur. Respir. J..

[37] Dweik R.A., Boggs P.B., Erzurum S.C., Irvin C.G., Leigh M.W., Lundberg J.O., Olin A.C., Plummer A.L., Taylor D.R. (2011). An official ATS clinical practice guideline: Interpretation of exhaled nitric oxide levels (FENO) for clinical applications. Am. J. Respir. Crit. Care Med..

[38] Katsardis C.V., Alexandraki S., Paraskakis E., Katsardis C., Koumbourlis A., Anthracopoulos M., Paraskakis E. (2015). Chapter 2: Spirometry in children 6-16 years old. Paediatric Pulmonary Function Testing Indications and Interpretation.

[39] Cole T.J., Lobstein T. (2012). Extended international (IOTF) body mass index cut-offs for thinness, overweight and obesity. Pediatr. Obes..

[40] IDS 25-Hydroxy Vitamin D ELISA Immuno Assays. https://www.idsplc.com/products/25-hydroxy-vitamin-ds-eia/.

[41] Holick M.F., Binkley N.C., Bischoff-Ferrari H.A., Gordon C.M., Hanley D.A., Heaney R.P., Murad M.H., Weaver C.M. (2011). Evaluation, treatment, and prevention of vitamin D deficiency: An Endocrine Society clinical practice guideline. J. Clin. Endocrinol. Metab..

[42] ISSAC Environmental Questionnaire 6–7 Years (ISAAC PHASE 3). https://isaac.auckland.ac.nz/phases/phasethree/environmentalquestionnaire/environmentalquestionnaire.html.

[43] Holick M.F. (2007). Vitamin D deficiency. N. Engl. J. Med..

[44] Serra-Majem L., Ribas L., Ngo J., Ortega R.M., García A., Pérez-Rodrigo C., Aranceta J. (2004). Food, youth and the Mediterranean diet in Spain. Development of KIDMED, Mediterranean Diet Quality Index in children and adolescents. Public Health Nutr..

[45] Jat K.R., Khairwa A. (2017). Vitamin D and asthma in children: A systematic review and meta-analysis of observational studies. Lung India.

[46] Liu J., Dong Y.-Q., Yin J., Yao J., Shen J., Sheng G.-J., Li K., Lv H.-F., Fang X., Wu W.-F. (2019). Meta-analysis of vitamin D and lung function in patients with asthma. Respir. Res..

[47] Chinellato I., Piazza M., Sandri M., Peroni D., Piacentini G., Boner A.L. (2011). Vitamin D serum levels and markers of asthma control in Italian children. J. Pediatr..

[48] Kerley C.P., Elnazir B., Faul J., Cormican L. (2015). Vitamin D as an adjunctive therapy in asthma. Part 1: A review of potential mechanisms. Pulm. Pharmacol. Ther..

[49] Pearce C., Jochmann A., Bush A., Horne R., Fleming L. (2016). Patterns of nonadherence in children with severe asthma. Eur. Respir. J..

[50] Ruiz-Ojeda F.J., Anguita-Ruiz A., Leis R., Aguilera C.M. (2018). Genetic Factors and Molecular Mechanisms of Vitamin D and Obesity Relationship. Ann. Nutr. Metab..

[51] Willeboordse M., van de Kant K.D.G., Tan F.E.S., Mulkens S., Schellings J., Crijns Y., Ploeg L.v.d., van Schayck C.P., Dompeling E. (2016). A Multifactorial Weight Reduction Programme for Children with Overweight and Asthma: A Randomized Controlled Trial. PLoS ONE.

[52] Ekström S., Hallberg J., Kull I., Protudjer J.L.P., Thunqvist P., Bottai M., Gustafsson P.M., Bergström A., Melén E. (2018). Body mass index status and peripheral airway obstruction in school-age children: A population-based cohort study. Thorax.

[53] Forno E., Weiner D.J., Mullen J., Sawicki G., Kurland G., Han Y.Y., Cloutier M.M., Canino G., Weiss S.T., Litonjua A.A. (2017). Obesity and Airway Dysanapsis in Children with and without Asthma. Am. J. Respir. Crit. Care Med..

[54] Boeke C.E., Oken E., Kleinman K.P., Rifas-Shiman S.L., Taveras E.M., Gillman M.W. (2013). Correlations among adiposity measures in school-aged children. BMC Pediatr..

[55] Hackshaw A. (2008). Small studies: Strengths and limitations. Eur. Respir. J..

[56] Jukic A.M.Z., Hoofnagle A.N., Lutsey P.L. (2018). Measurement of Vitamin D for Epidemiologic and Clinical Research: Shining Light on a Complex Decision. Am. J. Epidemiol..

[57] WHO (2010). WHO Guidelines on Drawing Blood: Best Practices in Phlebotomy. Chapter 6: Paediatric and Neonatal Blood Sampling.

[58] Immunodiagnostic Systems (IDS) Inc. (2019). OCTEIA 25-Hydroxy-Vitamin D. http://www.idsltd.com.

[59] DEQUAS (2019). Vitamin D External Quality Assessment Scheme. Vitamin D External Quality Assessment Scheme. http://www.deqas.org/.

