# Supplementary material for: Does BMI Modify the Association between Vitamin D and Pulmonary Function in Children of the Mild Asthma Phenotype?
Source: Int J Environ Res Public Health. 2022 Dec 14;19(24):16768. doi: 10.3390/ijerph192416768 (PMC9779255; doi:10.3390/ijerph192416768)
Supplement: Supplementary file 1 [file ijerph-19-16768-s001.zip › Supplemental S1 METHODS.pdf]

SUPPLEMENTAL S1 (METHODS)

MATERIALS AND METHODS

Determination of serum vitamin D concentrations

Venous samples (4ml) were collected from subjects according to WHO guidelines following a 2 hour fast. [1] The samples were centrifuged, plasma was decanted from the supernatant; they were stored at -20°C until analysis within 24 h to avoid degradation. In the case of hemolysis, blood collection was repeated. Vitamin D status was measured using serum 25-OH D as it has a half-life of 2-3 weeks and is the best indicator of vitamin D exposure from diet and dermal synthesis. Determination of 25-OH Vitamin D from blood serum was undertaken using Enzyme-Linked Immuno Assay (ELISA) (IDS, Tyne & Wear, UK). [2] A sample volume of 25µL was added to glass tubes, followed by 1mL of 25D-biotin conjugate, and the mixture vortexed. Then, 200µL of this mixture was added to the anti-25D antibody-coated microtitre plate. The assay mixture was incubated for 2 hours at room temperature, and plates were washed to remove unbound 25-D biotin conjugate. Bound 25-D biotin conjugate was identified by adding 200µL Avidin HRP (Horseradish peroxidase) for 30 minutes, and then the plate was washed again. Tetramethylbenzidine (TMB) substrate was added, followed by incubation for 30 minutes, and the reaction was terminated with acid. The absorbance recorded at 450 nm was inversely proportional to the concentration of 25-OH vitamin D. Serum 25(OH) D values were estimated for unknown samples directly from the calibration curve. The variability within and between assays was less than 1.9% and 4.6%, respectively. The assay's performance for the quantification of 25(OH)D was evaluated in collaboration with Vitamin D External Quality Assessment Scheme (DEQAS) in the UK. [3] Serum 25(OH)D is considered the best circulating biomarker of vitamin D metabolic status because it reflects dietary and sun exposure contributions. [4] We classified serum 25(OH)D levels ≥ 30 ng/mL as "sufficient", 20 to 30 ng/mL "insufficient", and < 20 ng/ml "deficient" based on previous recommendations. [4]

REFERENCES

1. WHO. WHO Guidelines on Drawing Blood: Best Practices in Phlebotomy. In Chapter 6: Paediatric and neonatal blood sampling. . Geneva: World Health Organization; 2010.

2. IDS. OCTEIA 25-Hydroxy- Vitamin D. Immunodiagnostic Systems (IDS) Inc. 2019. http://www.idsltd.com

3. DEQUAS. Vitamin D External Quality Assessment Scheme. Vitamin D External Quality Assessment Scheme. 2019. http://www.deqas.org/

4. Holick MF, Binkley NC, Bischoff-Ferrari HA, et al. Evaluation, treatment, and prevention of vitamin D deficiency: an Endocrine Society clinical practice guideline. *J Clin Endocrinol. Metab*. 2011;96(7):1911-30. doi:10.1210/jc.2011-0385
