# Supplementary material for: Does BMI Modify the Association between Vitamin D and Pulmonary Function in Children of the Mild Asthma Phenotype?
Source: Int J Environ Res Public Health. 2022 Dec 14;19(24):16768. doi: 10.3390/ijerph192416768 (PMC9779255; doi:10.3390/ijerph192416768)
Supplement: Supplementary file 1 [file ijerph-19-16768-s001.zip › Supplemental Table S1.pdf]

ONLINE SUPPLEMENT

SUPPLEMENTAL (Table S1)

Table S1. Population characteristics per sex.

Variables	Males (n= 30) Mean (SD)	Females (n= 31) Mean (SD)	P[a]
Age (years)	7.93 (2.33)	8.10 (2.21)	0.85[b]
BMI (kg/m^2)	19.00 (3.87)	18.43 (3.65)	0.49[b]
BMI categories			
Normal weight	60.0%(18)	54.8%(17)	0.68[c]
Overweight/Obese	40.0%(12)	45.2%(14)	
Regular exercise (≥ 3x/week) % (n)	53.3%(16)	45.2%(14)	0.52[c]
Medication therapy : Yes % (n)	76.7%(23)	87.1%(27)	0.29[c]
**PFTs**			
%FVC*	95.93 (10.52)	95.23 (9.07)	0.78
%FEV$_1$	99.33 (9.63)	97.29 (10.22)	0.42
% FEV$_1$/FVC	103.03 (5.83)	101.29 (6.30)	0.43[b]
% PEF	94.73 (14.65)	90.61 (18.51)	0.19[b]
% FEF$_{25-75\%}$	104.40 (17.55)	99.74 (21.42)	0.36
FeNO (ppb)	13.10 (14.70)	15.13 (13.39)	0.35[b]
25(OH) D (ng/mL)	24.95 (8.86)	21.38 (4.63)	0.09[b]
25(OH) D levels			
< 20 ng/mL (deficient) % (n)	20.0%(6)	38.7% (12)	0.09[c]
20-30 ng/mL (insufficient) % (n)	63.3%(19)	58.1%(18)	
≥ 30 ng/mL (sufficient) % (n)	16.7%(5)	3.23%(1)	

Total N = 61

Continuous variables presented as mean (SD) and categorical, as percentage % (n)

[a]P- P-value estimated using T-test; [b] Mann-Whitney U test; [c] Chi Square test

*Percent predicted pre-bronchodilator administration

Key: PFTs- Pulmonary Function Tests, FEV$_1$- Forced Expiratory Volume in 1 second, FVC- Forced Vital Capacity, FEV$_1$/FVC- ratio of Forced Expiratory Volume in 1 second and Forced Vital Capacity, PEF- Peak Expiratory Flow, FEF $_{25-75\%}$ -Forced Expiratory Flow at 25-75% of the pulmonary volume, FeNO- Fractional exhaled Nitric Oxide
